# Bifunctional Lipid–Protein Cross-linking Efficiency and Reaction Products

**DOI:** 10.1021/jacs.6c01205

**Published:** 2026-04-10

**Authors:** Carla Kirschbaum, H. Mathilda Lennartz, Katelyn C. Cook, Kristin Böhlig, Athanasios Papangelis, Carol V. Robinson, André Nadler

**Affiliations:** † Kavli Institute for Nanoscience Discovery, 6396 University of Oxford, Oxford OX1 3QU, United Kingdom; ‡ Department of Chemistry, 6396 University of Oxford, Oxford OX1 3QZ, United Kingdom; § Max Planck Institute of Molecular Cell Biology and Genetics, 01307 Dresden, Germany; ⊥ Cluster of Excellence Physics of Life, TU Dresden, 01307 Dresden, Germany

## Abstract

Bifunctional diazirine lipids are versatile tools for mapping protein–lipid interactions and cellular localization by photo-cross-linking. Yet, the cross-linking efficiency of these probes has not been systematically evaluated. We use the lipid transfer protein STARD10, which binds phospholipids in a 1:1 stoichiometry within a hydrophobic pocket, to measure the upper limit of the photo-cross-linking efficiency of bifunctional lipid probes. We characterize reaction products using native and denaturing mass spectrometry. Our results show that approximately 5% of photoactivated lipids form covalent protein–lipid cross-links, while the majority follow intramolecular reaction trajectories, resulting in the formation of products featuring alkene, ketone and hydroxyl moieties. These findings provide essential context for the use of bifunctional probes to uncover the cell biology of lipids and highlight the need for continuous improvement to experimental workflows.

## Graphical abstract

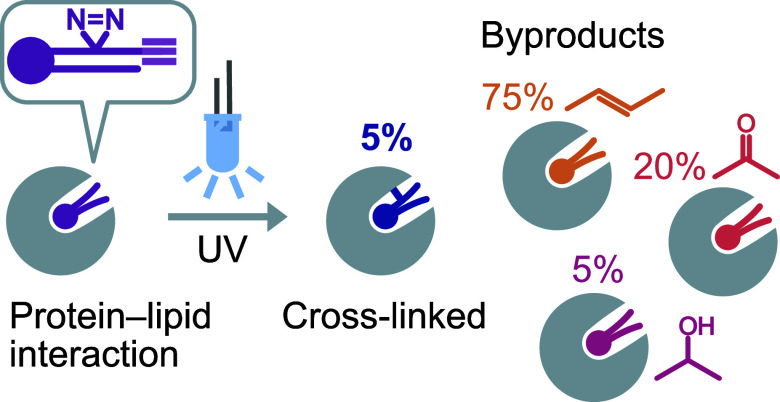

Diazirine photoreactive groups are widely used to investigate protein–protein and protein–small molecule interactions by photoaffinity labeling.^1–5^ Diazirine-containing phospholipids closely mimic the behavior of natural lipids and readily mix with endogenous membranes, making them particularly valuable for probing protein–lipid interactions.^6,7^ Bifunctional lipids that combine a diazirine moiety with an alkyne tag have emerged as powerful probes for mapping lipid interactomes^8–16^ and are currently the most reliable tools for monitoring species-specific intracellular lipid localization and transport at high resolution.^17–21^

The reactivity of dialkyl diazirines has become increasingly well understood in recent years. Upon UV-A irradiation, they generate highly reactive carbenes through the expulsion of N_2_, but also form substantial amounts of diazo intermediates (cf. Figure 1A).^22,23^ Those can either convert into carbenes or react directly with proteins.^22^ Unlike carbenes, diazo intermediates have a chemical lifetime above the diffusion limit^24^ and react over a wider radius, with a high selectivity for acidic and polar amino acids.^25–28^ Together, the rapid, diffusion-limited carbene pathway and the slower diazo-mediated pathway shape the final product distribution.^23^

**Figure 1:**
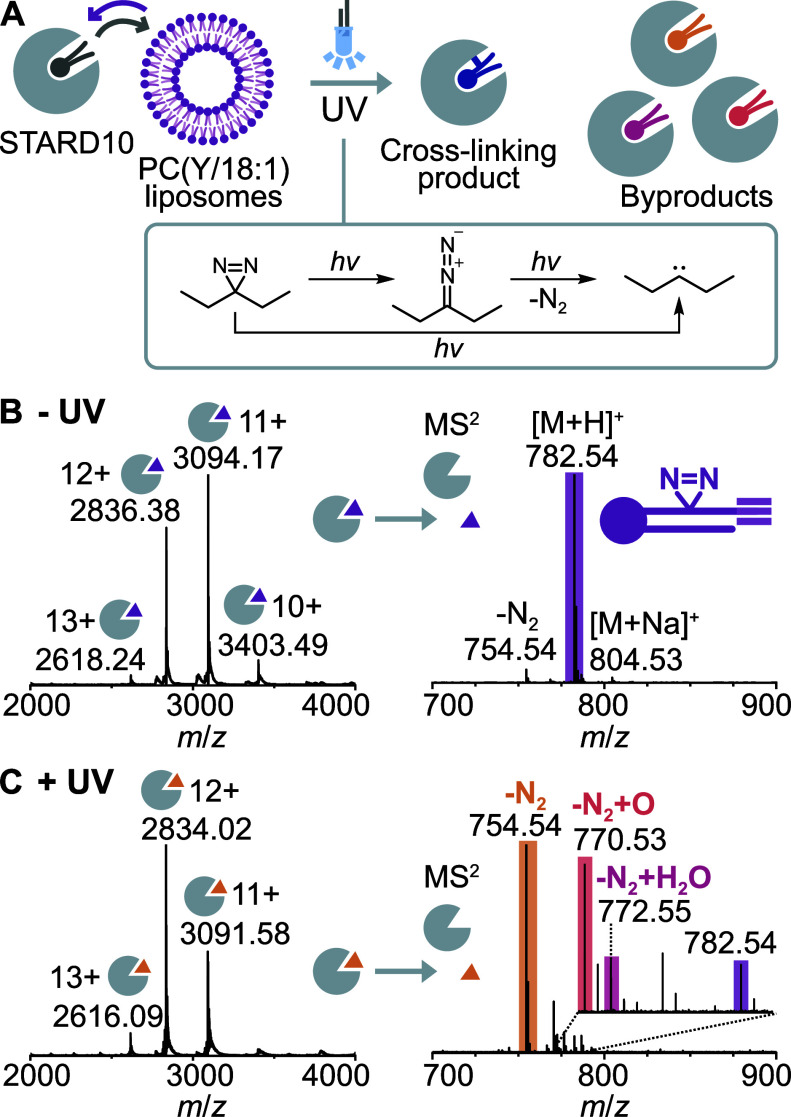
Cross-linking of STARD10 and bifunctional PC *in vitro*. (A) STARD10 is incubated with liposomes containing 80% PC­(Y/18:1) and 20% cholesterol to replace copurified bacterial lipids (dark gray) with diazirine PC (purple). Diazirine PC forms reactive diazo and carbene intermediates upon UV irradiation. (B) Native MS before photoactivation confirms saturation of the 1:1 STARD10:PC­(Y/18:1) complex. Colored triangles represent bound lipids. (C) Native MS of STARD10 and release of non-cross-linked lipids after UV irradiation shows nitrogen loss products and a minor fraction (ca. 25%) of oxidized lipids and unreacted lipid probe (ca. 5%). Additional peaks corresponding to sodium adducts are not labeled for clarity.

The efficiency of the protein–lipid cross-linking reaction remains undefined, despite the fact that it is one of the key experimental constraints when studying lipid localization and lipid–protein interactions. Model peptides have shown cross-linking rates of up to 100% when exposed to excess diazirine reagent, but these rates drop to nearly zero in aqueous solution.^22^ In 2:1 phosphatidylcholine (PC):diazirine-PC liposomes containing model transmembrane peptides, ca. 1–10% peptide–lipid cross-linking products were recovered after repeated irradiation,^6,7^ but those values are highly system-dependent. It is well-established that the cross-linking efficiency is significantly reduced by intra- and intermolecular side reactions of the reactive intermediates. Notably, intramolecular alkene formation of photoactivated diazirines can account for >50% of the reaction products,^23^ and lipid–lipid cross-linking is frequently observed in liposomes.^6,7^ We sought to determine the upper limit of lipid–protein cross-linking efficiency and the extent of the competing intramolecular reaction pathways, while reducing side-reactions with other lipids and small molecules. Choosing a well-defined model system for this purpose is essential, as the vast number of possible cross-linking products in cellular assays makes reliable quantification of overall cross-linking yields impossible.

We therefore chose the soluble lipid transfer protein STARD10 and bifunctional PC derivatives as a model system. STARD10 binds PC in a 1:1 stoichiometry within a hydrophobic binding pocket that excludes water.^29^ Hence, any lipid molecule activated inside this pocket can, in principle, undergo only two possible reactions: cross-linking to the protein or internal rearrangement. As STARD10 forms a 1:1 complex with the respective lipid probe, the relative fraction of covalent protein–lipid conjugates and rearranged lipid products can be directly determined under ideal, solvent-free conditions.

We used native and denaturing mass spectrometry (MS) to characterize the reaction products and quantify the cross-linking efficiency after a 3-s irradiation pulse with 365 nm high-power LEDs. For STARD10 saturated with bifunctional PC, we observed a cross-linking yield of approximately 5%, whereas the majority of bound lipids formed alkenes, and a minor fraction resulted in oxidation products. Our results suggest that a significant loss of signal-to-noise ratio during the photo-cross-linking step should be taken into account when planning experiments with bifunctional lipids.

We obtained human STARD10 by recombinant expression in *E. coli.* ^100^ The protein copurified with bacterial phospholipids in a 1:1 stoichiometry, as confirmed by native MS (Figure S1). To replace the natural phospholipids with diazirine lipids, we incubated purified STARD10 with liposomes consisting of 80% bifunctional PC­(Y/18:1) (full structure shown in Figure 3A) and 20% cholesterol in PBS solution (Figure 1A). After 10 min incubation at room temperature, we removed excess lipids by buffer exchange into ammonium acetate (200 mM, pH 7) for native MS.

The native mass spectrum of STARD10 incubated with PC­(Y/18:1) showed a charge state distribution from 10–13+ corresponding by mass to STARD10 bound to PC­(Y/18:1) in a 1:1 stoichiometry (Figure 1B). To confirm the identity of bound lipids, we isolated the protein–lipid complex in the ion trap and used collisional activation to release noncovalently bound lipids from STARD10. The MS^2^ spectrum confirmed that the bound lipids corresponded to intact PC­(Y/18:1). A minor peak indicated partial nitrogen loss, which we ascribe to the collisional activation. Overall, the MS data confirmed that the diazirine lipids had entirely replaced the bacterial phospholipids copurified with STARD10, providing ideal conditions for assaying cross-linking efficiency and reaction products.

We next irradiated the 1:1 STARD10:PC­(Y/18:1) complex for 3 s using a high-powered 365 nm LED. Under these conditions, the photoactivation reaction is 95% complete (Figure 1C), largely in line with light-dose response experiments *in vitro* and in cells, which we conducted previously with the same setup.^19^ After photoactivation, we removed excess lipids by buffer exchange and investigated the reaction products by native MS. Consistent with the expected loss of the diazirine group upon UV photoactivation, we observed a decrease in the mass of the protein–lipid complexes relative to the control without UV irradiation (Figure 1C). To characterize the reaction products, we isolated the protein–lipid complex in the gas phase and released noncovalently bound lipids by collisional activation. The majority of reaction products (ca. 75%) corresponded by mass to the loss of N_2_ (−28 Da), whereas additional peaks shifted by +16 and +18 Da suggested partial lipid oxidation (ca. 25%). The original diazirine-containing probe made up only 5% of the bound lipids after photoactivation, suggesting that it was almost entirely consumed within the 3-s irradiation pulse.

Noncovalent protein–lipid complexes resulting from alkene formation are indistinguishable by mass from covalent protein–lipid cross-linking products (Figure 2A), rendering native MS unsuitable for determining the fraction of STARD10 that was efficiently cross-linked. To disrupt noncovalent protein–lipid interactions while preserving covalently bound lipids, we denatured the protein in organic solvent (45% acetonitrile, 5% isopropanol, 1% formic acid). Denaturing MS of STARD10 incubated with PC­(Y/18:1) in the absence of UV irradiation yielded a broad charge state distribution from 10–41+ corresponding to the unmodified apo protein (Figure 2B). Deconvolution of the spectrum confirmed that only negligible amounts of lipid remained associated with the protein after denaturation (ca. 1%), demonstrating effective removal of non-cross-linked lipids.

**Figure 2:**
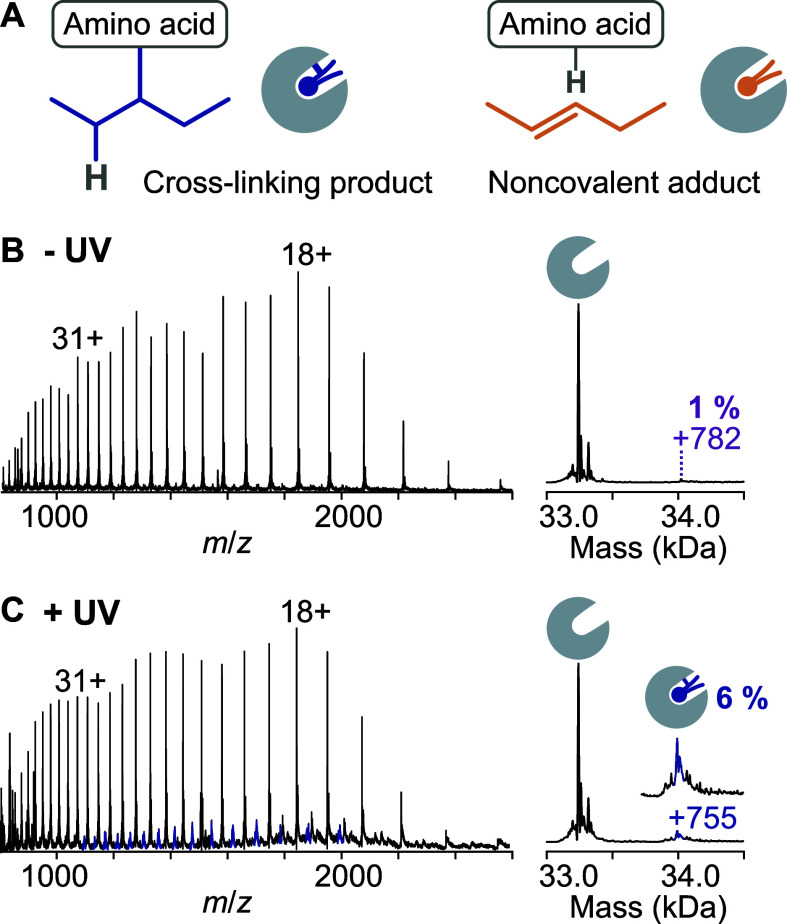
Cross-linking efficiency determined by denaturing MS. (A) Covalent protein–lipid complexes are indistinguishable by mass from noncovalently bound alkene byproducts. (B) Denaturing MS of STARD10 incubated with PC­(Y/18:1) without UV irradiation confirms disruption of noncovalent protein–lipid interaction. (C) Denaturing MS of STARD10 incubated with PC­(Y/18:1) and cross-linked with UV light reveals that photoactivation led to cross-linking in ca. 5% of the cases, assuming 1% residual noncovalent binding.

To quantify the protein–lipid cross-linking efficiency, we performed denaturing MS of STARD10 incubated with PC­(Y/18:1) and irradiated with UV light (Figure 2C). Besides the major charge state distribution corresponding to the unmodified apo protein, the mass spectrum featured a minor charge state distribution corresponding to STARD10 covalently bound to one PC molecule. We deconvoluted the spectrum using UniDec^30^ and determined by peak integration that ca. 6% of the protein was cross-linked to PC at a 1:1 ratio. When accounting for 1% of nonspecific lipid binding (Figure 2B), the estimated cross-linking efficiency amounts to approximately 5%. This ratio was consistent across technical replicates (Figure S2). Because the protein was saturated with PC­(Y/18:1) prior to UV irradiation and the precursor was almost entirely consumed after irradiation, we conclude that ca. 5% of the photoactivated lipid resulted in effective cross-linking, whereas the majority (approximately 70%) led to nitrogen loss and intramolecular rearrangement without cross-linking to an amino acid.

To consolidate these values, we also quantified the cross-linking efficiency of bifunctional PC probes with alternative side chains (PC­(Y/16:0) and PC­(Y/20:4)), which could influence how the lipid chains are folded inside the STARD10 lipid-binding pocket (Figure S3). Like PC­(Y/18:1), both lipids were readily loaded onto STARD10 via liposomes, though PC­(Y/16:0) replaced the bacterial lipids less effectively than the unsaturated lipid probes. Denaturing MS revealed similar cross-linking rates between 5–7% for PC­(Y/16:0) and PC­(Y/20:4), suggesting high reproducibility of the photoreaction pathways.

To further characterize the byproducts formed upon photoactivation that do not lead to covalent protein–lipid cross-linking, we measured ultraviolet photodissociation (UVPD) mass spectra of all reaction products (Figure 3). UVPD is an ion activation technique that induces cleavage of C–C bonds and thus yields deeper insight into lipid structure than classical collision-based fragmentation. Spectra were acquired in (+) mode to enhance sensitivity. This is due to the fact that electron photodetachment is a major reaction pathway in UVPD, which results in a neutral product molecule in (−) mode and relatively fewer fragment ions.^31^

**Figure 3:**
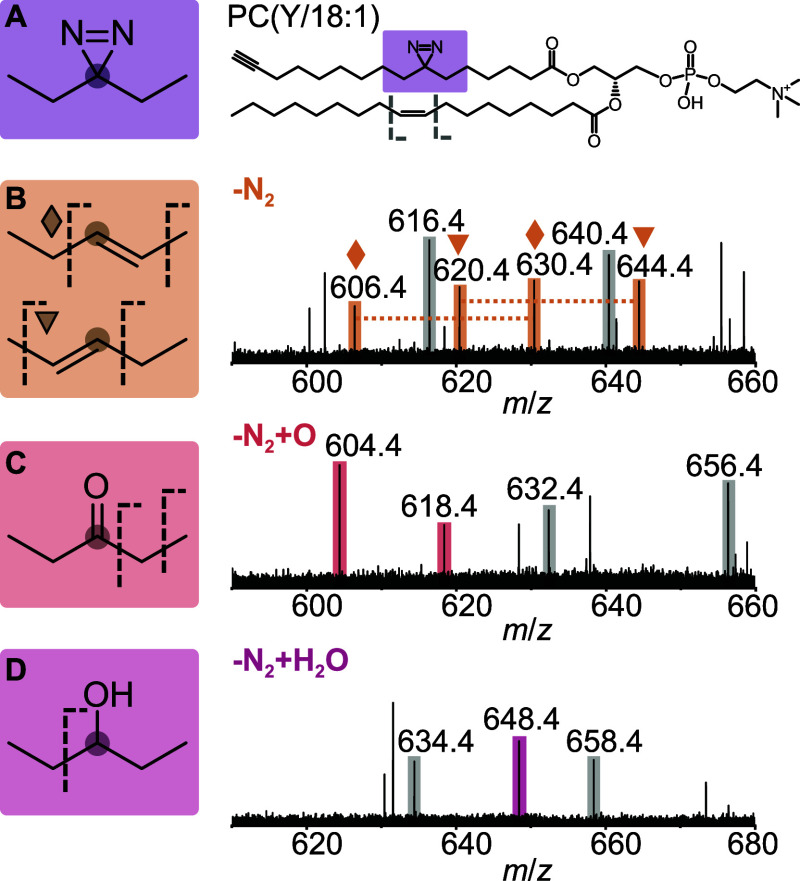
Characterization of byproducts by ultraviolet photodissociation mass spectrometry. Photoactivation of the diazirine lipid PC­(Y/18:1) (A) bound to STARD10 resulted in the formation of two regioisomeric alkenes (B), ketones (C) and alcohols (D) inserted at the original position of the diazirine moiety. Dotted lines indicate C–C bond cleavage by UVPD. Peaks labeled in gray are derived from the C=C bond in the 18:1 acyl chain. Spectra were acquired in (+) mode.

In the UVPD spectrum of the main byproduct corresponding by mass to the loss of N_2_, we detected two pairs of peaks shifted by 24 Da, which is indicative of alkene formation (Figure 3B).^32^ The fragment spectrum confirmed the formation of two alkene regioisomers with double bonds to the left and right carbon neighboring the original diazirine site. Note that the configuration (*E*/*Z*) of the newly formed C=C bond cannot be determined with this approach. The C=C bond in the native 18:1 acyl chain of the PC­(Y/18:1) probe yielded an additional pair of peaks shifted by 24 Da (shown in gray). Based on the relative intensities of the fragment ions, we suggest that both alkene regioisomers are generated in comparable amounts.

The oxidized lipid products correspond most likely to a ketone (+16 Da) (Figure 3C) and an alcohol (+18 Da) (Figure 3D) inserted at the original diazirine site. For the ketone, we observed a fragment resulting from cleavage between the alpha and beta carbon, a reaction that has previously been observed for small molecules containing ketones.^33^ A minor fragment was also formed by C–C bond cleavage adjacent to the carbonyl oxygen. The alcohol yielded a diagnostic fragment that contained the newly formed OH group by cleavage of the C–C bond adjacent to the original diazirine site. Full fragment ion assignments supported by high-resolution Orbitrap mass spectra can be found in Figure S4 and Table S1. Overall, we observed ca. 75% alkene, 20% ketone and 5% alcohol formation in the original diazirine acyl chain.

The byproducts identified by MS are consistent with previous studies. It is known that alkene formation is the major side reaction of photoactivated dialkyl diazirines in liposomes and nonaqueous solutions.^7,23^ Under aqueous conditions (80% water), photoactivation of a soluble dialkyl diazirine yielded a mixture of 50% alkene, 10–20% ketone and 35% alcohol through water insertion.^28^ The reduced abundance of oxidized lipids observed in our experiment is reasonable because the diazirine lipids are integrated into the hydrophobic binding pocket of STARD10, minimizing their exposure to water.

The average cross-linking efficiency of 5% determined in this study is within the same order of magnitude as previous studies reporting 1–10% cross-linking efficiency using 300 or 365 nm irradiation,^6,7^ although these values cannot be directly compared due to varying experimental conditions such as light source, irradiation time, and initial protein and diazirine lipid concentrations. Our results imply that 5% cross-linking yield represent a practical upper limit that can be achieved in the case of a well-defined 1:1 protein:lipid complex featuring a tightly bound lipid within a geometrically confined binding pocket. This allows for the more general conclusion that a large majority of diazirine phospholipids will not form cross-links to proteins in cellular assays. A larger number of protein–lipid cross-links is expected statistically for proteins that interact with multiple lipids, such as membrane proteins which are preferentially labeled by dialkyl diazirine probes,^28^ though the intrinsic chemistry of each individual lipid probe remains unchanged.

This expected cross-linking yield needs to be taken into account for any assay development using diazirine lipids to predict experimental yield and necessary biochemical parameters. While a full discussion of the advantages and limitations of bifunctional lipids is beyond the scope of this manuscript (the reader is referred to recent publications^8–21^ on the subject), there are several conclusions to be drawn from the findings of this study. For instance, starting bifunctional lipid concentrations should be approximately 20 times higher compared to fluorescence lipid probes to achieve similar signal intensity in imaging assays, assuming that the upper boundary of lipid–protein cross-linking in cells is similar to or lower than observed here. To an extent, this can be mitigated by the choice of fluorescent dye used for labeling, but it also highlights the need for optimizing other experimental parameters such as UV light sources, where LEDs should be used instead of arc lamps, and reaction conditions for click chemistry derivatizations.

The main limitation of our study is its focus on a single lipid class (PC) and its protein binding partner, STARD10, which limits its generality. However, we have reason to believe that lipid–protein cross-linking yields are roughly comparable across lipid classes for probes featuring the diazirine in the middle of the aliphatic chain, as fluorescence intensities in lipid imaging experiments are primarily determined by lipid concentration in cells rather than probe type.^17^ In contrast, the reaction efficiency may change if the diazirine is placed closer to the headgroup. In fact, it was recently reported that diazirine placement does affect identified proteins in mass spectrometric lipid–protein interactions screens, indicating changes in cross-linking reactions,^34^ but it is yet unclear whether these changes are indicative of increased or decreased cross-linking efficiency.

We anticipate that our results will provide a valuable reference point for designing cross-linking studies based on diazirine lipids, which are increasingly used for mapping intracellular lipid localization, measuring lipid transport and identifying lipid–protein interactions.

## Supplementary Material



## Data Availability

Raw mass spectra are accessible via Figshare (DOI: 10.25446/oxford.30814682).

## Notes

Materials and methods, supplementary mass spectra and fragment assignments (PDF)
